# Associations of Occupational Stressors, Perceived Organizational Support, and Psychological Capital with Work Engagement among Chinese Female Nurses

**DOI:** 10.1155/2017/5284628

**Published:** 2017-01-12

**Authors:** Xiaoxi Wang, Li Liu, Futing Zou, Junhui Hao, Hui Wu

**Affiliations:** ^1^Department of Sport Medicine, School of Fundamental Sciences, China Medical University, No. 77 Puhe Road, Shenyang North New Area, Shenyang, Liaoning 110122, China; ^2^Department of Social Medicine, School of Public Health, China Medical University, No. 77 Puhe Road, Shenyang North New Area, Shenyang, Liaoning 110122, China

## Abstract

This study aimed to explore the associations of occupational stressors (extrinsic effort, reward, and overcommitment), perceived organizational support (POS), and psychological capital (PsyCap) and its components (self-efficacy, hope, resilience, and optimism) with work engagement and the mediating roles of PsyCap and its components among Chinese female nurses within the framework of the job demands-resources (JD-R) model. A cross-sectional sample (1,330) completed the Utrecht Work Engagement Scale, Effort-Reward Imbalance Scale, Survey of POS, and PsyCap Questionnaire, and effective respondents were 1,016 (76.4%). Hierarchical regression analysis and Preacher and Hayes' asymptotic and resampling strategies were used. Extrinsic effort was negatively associated with vigor, dedication, and absorption, while POS, PsyCap, and hope were positively associated with them. Reward and overcommitment were positively associated with dedication and absorption. Optimism was positively associated with vigor and dedication. Optimism mediated the associations of extrinsic effort, reward, and POS with vigor and dedication. PsyCap and hope mediated the associations of POS with vigor, dedication, and absorption. There is a low level of work engagement among Chinese female nurses. Extrinsic effort could reduce work engagement, while reward, overcommitment, POS, PsyCap, hope, and optimism could enhance work engagement. Hospital managers should develop the PsyCap of female nurses through controlling occupational stressors and establishing supportive organizational climate to enhance their work engagement.

## 1. Introduction

Work engagement is defined as a positive, fulfilling, and work-related state of mind. As the antithesis of job burnout, work engagement consists of vigor, dedication, and absorption [1]. The focus of work engagement is on strengths rather than weaknesses in work in the field of positive psychology. In recent years, as an important influence factor of individual's mental health and positive organizational behavior, work engagement has attracted the attention from various fields including education, business, and healthcare services [2–4]. In nursing services, previous studies have reported that high level of work engagement can enhance nurses' job performance, satisfaction, and emotional health and reduce their turnover intention [4–7]. Meanwhile, work engagement has a positive effect on the attitudes of nurses towards patients [8]. Thus, a low level of work engagement not only adversely affects patients' health but also deteriorates the quality of nursing services. Along with the development of medical and health services in China, an increasingly higher standard is placed on the quality of nursing service. At present, China has a density of nursing and midwifery personnel per 1,000 people at 1.656, which is lower than that in developed countries and the majority of developing countries in the world [9]. Therefore, given the enormous service population in China, it is of great importance to explore the influence factors of work engagement among nurses.

Recent studies on worker mental health have shifted their focus from psychological distress to positive emotions at work, especially on work engagement. Work engagement has frequently been studied within the framework of the job demands-resources (JD-R) model. This model is an overarching model, which combines positive and negative outcomes of employee health and wellbeing. Therefore, the model not only integrates related previous models, such as Effort-Reward Imbalance (ERI) model, but also compounds two separate research traditions, namely, the “stress research tradition” and the “motivational research tradition.” Nevertheless, the previous models mainly focused on the negative outcomes of job strain (stress research tradition). The JD-R model is good to make up this flaw and is also proven to be useful in the conceptualization of wellbeing, work engagement, and performance [10, 11].

According to the JD-R model, working conditions can be classified into two general categories (i.e., job demands and job resources) that are applicable to virtually all occupations. Basically, job demands require effort and related with physiological and psychological costs, whereas job resources foster personal growth, learning, and development and have motivational qualities by a wide range of resources at work, including job resources (e.g., job control, rewards at work, social support, and organizational support at work) and personal resources (e.g., psychological capital). The JD-R model distinctly explains the different interactions between job resources, personal resources, and job demands. Work engagement is the result of intricate interactions. The combination of job resources, personal resources, and job demands has to operate in such a way as to provide employees with the opportunity to experience engagement. Job and personal resources, considered respectively and in combination, predict work engagement [12, 13].

Work engagement is significantly related to individual and organizational factors [5, 14]. Occupational stress is a complex biopsychosocial condition and has become a wide public concern in workplaces. There is high prevalence of occupational stress among nurses in general, with studies from different countries reporting the prevalence from 24.5% to 44.2% [15–19]. Among Chinese nurses in particular, the prevalence of occupational stress was about 25% to 40% [15, 16]. Occupational stress refers to the response employees may have when they are confronted with work demands and pressures that are not matched by their knowledge and abilities and challenge their ability to cope. The stress can be caused by various occupational stressors from poor work organization, design and management, and unsatisfactory working conditions. As human healthcare professionals, nurses are highly exposed to various occupational stressors such as work overload, role conflict, effort-reward imbalance, and unsatisfactory nurse-patient relationship [20–22]. Unfortunately, the level of occupational stress has been increasing in China because of the limited nursing workforce and healthcare system reform. Occupational stress can result in various negative organizational behaviors and health outcomes in nurses. There are many researches that have found positive relationships between occupational stress and job burnout in nurses across countries [23–25]. Occupational stress has been identified as a predictor of work engagement in the workers of a metallurgical industry [26]. Also, as occupational stressors, workload and time pressure showed a positive association with work engagement among Japanese employees [27]. Fiabane et al. reported that workload could decrease the work engagement in nurses and other healthcare workers [28]. The contradictory finding might be caused by different levels of occupational stressors in different groups. As a challenge at work, higher levels of workload and time pressure may enhance work engagement, but they may also reduce it as a hindrance. As a protective factor, reward can be positively related to nurses' work engagement [5, 29].

The Effort-Reward Imbalance (ERI) model is often used to measure occupational stress in workplaces. The ERI model claims that work characterized by both high extrinsic efforts and low rewards represents a reciprocity deficit between high “costs” and low “gains,” which could elicit the negative emotions of employees [30–32]. Another assumption of the ERI model is that employees have a motivational pattern of excessive job-related commitment (overcommitment). The ERI model has been shown to be suitable for evaluations of adverse health effects caused by occupational stress among hospital nurses in China on individual level. This manuscript aimed to analyze the association of occupational stressors (extrinsic effort, reward, and overcommitment) with work engagement in Chinese nurses.

According to organizational support theory, perceived organizational support (POS) refers to employees' general beliefs regarding the extent to which “the organization values their contributions and cares about their wellbeing” [33]. Since supervisor and department are often viewed as organizational agents, POS is a construct distinct from perceived supervisor or department support [34]. POS can increase employees' inner obligation to help the organization reach its objectives and their affective commitment to the organization. Therefore, POS can increase employees' in-role and extra-role performance and decrease their stress and withdrawal behaviors. Empirical researches have indicated that POS has positive impacts on various organizational behaviors and health benefits in nurses. Mahon et al. reported that POS had a direct, positive association with employee engagement, with data from 231 team members of two organizations [35]. Although the relationship between POS and job burnout has been confirmed in nurses [36, 37], the potential impact of POS on work engagement has not been examined in this occupational population to our best knowledge.

Personal resources had been defined as “positive self-evaluations that are linked to resiliency and refer to individuals' sense of their ability to control and impact upon their environment successfully.” Personal resources, such as psychological capital (PsyCap), have empirically been shown to predict work engagement. PsyCap is a positive exploitable psychological state that individual performs during growth and development processes [38]. There are four components constituting PsyCap, including self-efficacy, hope, resilience, and optimism. PsyCap has significantly positive effects on employee's performance, satisfaction, and wellbeing [39]. In recent years, many studies were carried out to explore the positive effects of PsyCap on work behaviors and occupational health outcomes in nurses worldwide, and job burnout was one of the important research outcomes. PsyCap not only can directly reduce the level of burnout [40–42] but also can act as a mediator in the association between work-family conflict and burnout in nurses [42]. This suggests that PsyCap might be an effective personal resource to improve work engagement [43–45]. However, the types of effects (direct or mediating effects) of PsyCap and the antecedent variables of PsyCap are different across occupational groups. According to the results of previous studies, both POS and occupational stressors could affect the level of PsyCap in different occupational groups. In other words, POS and occupational stressors are the antecedents of PsyCap. As a result, PsyCap and its components may act as mediators in the relationships between POS, occupational stressors, and work engagement.

However, few studies estimated the mediating role of PsyCap on the relationship among job demand, job resources, and work engagement in Chinese nurses. Resolving the questions might contribute to further understanding the role of PsyCap for developing a workplace intervention. In light of the above concerns, using the JD-R model, the present study aimed to verify the following three hypotheses among Chinese female nurses: (1) extrinsic effort, reward, overcommitment, and POS were associated with PsyCap and its four components (self-efficacy, hope, resilience, and optimism); (2) extrinsic effort, reward, overcommitment, POS, and PsyCap and its components were associated with the three components of work engagement (vigor, dedication, and absorption), respectively; (3) PsyCap and its components mediated the associations of extrinsic effort, reward, overcommitment, and POS with vigor, dedication, and absorption, respectively.

## 2. Materials and Methods

### 2.1. Ethics Statement

The study was conducted in accordance with the Declaration of Helsinki, and the protocol was approved by the Committee on Human Experimentation of China Medical University. Written informed consent was obtained from the participants in this study who voluntarily participated in the study. We protected the privacy and anonymity of individuals involved in our research.

### 2.2. Subjects and Data Collection

A cross-sectional study was conducted in Shenyang, Liaoning province, from October to November 2014. The city comprises thirteen districts, and five districts were selected in this study. We randomly selected two large general hospitals (>500 beds) from each selected district. Thus, a total of ten large general hospitals were included in the present study. Due to the extreme low percentage of male nurses in China, only female nurses were our research focus. In these selected hospitals, female nurses who were certificated and engaged in nursing work for more than 12 months became our subjects. After obtaining a written informed consent from each participant, self-administered questionnaires were directly distributed to 1,330 nurses from the departments of internal medicine, surgery, obstetrics and gynecology, pediatrics, laboratory, radiology, emergency, ICU, and oncology, and they were completed anonymously in a private room after the respondent's shift was over. Complete responses were obtained from 1,016 participants (effective response rate: 76.4%). The average age of the subjects was 33.6 (SD = 8.7) years, and the average organizational tenure was 12.6 (SD = 9.5) years.

### 2.3. Measurement of Work Engagement

Work engagement was evaluated using the 9-item version of the Utrecht Work Engagement Scale (UWES) [1]. The UWES has three dimensions including vigor (3 items), dedication (3 items), and absorption (3 items), which are described as the three defining attributes of work engagement. All items are scored on a 7-point Likert scale ranging from 0 (never) to 6 (every day), and the average score of each dimension was calculated. A higher score indicates a higher level of work engagement. The Chinese version of the UWES has been used in Chinese occupational groups, and it has satisfactory reliability and validity [46, 47]. In the present study, Cronbach's alpha coefficients for vigor, dedication, and absorption subscales were 0.78, 0.84, and 0.80, respectively.

### 2.4. Measurement of Occupational Stressors

The Chinese version of the ERI Scale has been widely applied among Chinese occupational groups with good reliability and validity [48, 49]. The ERI Scale consists of three dimensions: extrinsic effort (6 items), reward (11 items), and overcommitment (6 items). All items of extrinsic effort and reward subscales are scored on a 5-point scale in which 1 indicates no stressful experience and 5 indicates the highest level of stressful experience. Higher scores indicate higher levels of extrinsic effort and reward. Each item of overcommitment is scored on a 4-point Likert scale in which 1 indicates complete disagreement and 4 indicates complete agreement with each statement. A higher score suggests higher demand characterized by excessive work-related commitment [50]. In this study, Cronbach's alpha coefficients for extrinsic effort, reward, and overcommitment subscales were 0.90, 0.81, and 0.74, respectively.

### 2.5. Measurement of POS

A short version of the Survey of Perceived Organizational Support (SPOS) was used to assess POS [33]. The scale has 9 items. A higher score indicates a higher POS. The short version of the SPOS has been widely applied among Chinese occupational groups with good reliability and validity [51, 52]. Cronbach's alpha coefficient for the POS scale was 0.88 in this study.

### 2.6. Measurement of PsyCap

The 24-item Psychological Capital Questionnaire (PCQ) was used to measure PsyCap [35]. The PCQ consists of four subscales: self-efficacy (6 items), hope (6 items), resilience (6 items), and optimism (6 items). All items are scored on a 6-point Likert scale in which 1 indicates strong disagreement and 6 indicates strong agreement. Higher values indicate higher level of PsyCap and its components. The Chinese version of the PCQ has been used in Chinese studies, and it has satisfactory reliability and validity [40–42, 48]. In this study, Cronbach's alpha coefficients for self-efficacy, hope, resilience, and optimism subscales and the total scale were 0.89, 0.88, 0.77, 0.74, and 0.93, respectively.

### 2.7. Demographic Characteristics

Demographic factors included age, marital status, and education. Marital status was categorized as “single/widowed/divorced/separated” and “married/cohabitated.” Education was categorized as “junior college or lower” and “college or higher.”

### 2.8. Statistical Analysis

**Figure 1:**
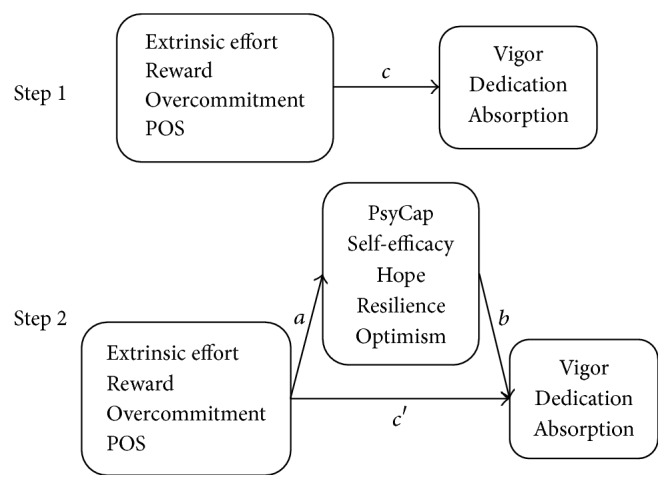
Theoretical model of the mediating roles of PsyCap and its components in the associations of extrinsic effort, reward, overcommitment, and POS with vigor, dedication, and absorption. (*c*) The associations of extrinsic effort, reward, overcommitment, and POS with vigor, dedication, and absorption; (*a*) the associations of extrinsic effort, reward, overcommitment, and POS with PsyCap and its components; (*b*) the associations of PsyCap and its components with vigor, dedication, and absorption after controlling for other independent variables; (*c*′) the associations of extrinsic effort, reward, overcommitment, and POS with vigor, dedication, and absorption after adding PsyCap or its components as mediators.

Comparisons on continuous variables were made by Student's* t*-test or one-way ANOVA. Correlations among continuous variables were examined using Pearson's correlation analysis. Hierarchical linear regression analysis was performed to examine the associations of extrinsic effort, reward, overcommitment, POS, and PsyCap and its four components with vigor, dedication, and absorption, respectively. In block 1, demographic variables (age, marital status, and education) were added. In block 2, extrinsic effort, reward, overcommitment, and POS were added. In block 3's model 1, PsyCap was added. In block 3's model 2, the four components of PsyCap were added. Variance inflation factor (VIF) was used to check for multicollinearity, and the values of VIFs suggested that multicollinearity was not a problem in the estimates presented in this study. Moreover, asymptotic and resampling strategies were used to examine PsyCap and its components as potential mediators in the associations of extrinsic effort, reward, overcommitment, and POS with vigor, dedication, and absorption, respectively [53]. Extrinsic effort, reward, overcommitment, and POS were modeled as independent variables, with vigor, dedication, and absorption as outcomes, PsyCap and its components as mediators (as shown in Figure 1), and age, marital status, and education as covariates. If the absolute value of path coefficient *c*′ in Step  2 is smaller than that of the path coefficient *c* in Step  1 or *c*′ is not statistically significant, the mediating roles of PsyCap or its components may exist. 5000 bootstrap samples were used to estimate parameters in this study. A bias-corrected and accelerated 95% confidence interval (BCa 95% CI) was determined for each *a* × *b* product, and a BCa 95% CI excluding 0 indicated a significant mediating role. Before performing the regression analyses, all continuous variables including the predictor variables and the mediator variables were centralized to account for differences in scale scores.

## 3. Results

### 3.1. Participant Characteristics

The demographic characteristics of subjects and comparisons on vigor, dedication, and absorption are shown in Table 1. The mean scores of vigor, dedication, and absorption were 3.21 (SD = 1.63), 3.44 (SD = 1.70), and 2.73 (SD = 1.70), respectively. The scores of vigor, dedication, and absorption in the age group of >40 years were significantly higher than those in ≤30 and 30–40 years groups, respectively (vigor: *F* = 10.913 and *P* < 0.01; dedication: *F* = 10.694 and *P* < 0.01; absorption: *F* = 13.367 and *P* < 0.01). Marital status was not significantly related to the scores of vigor, dedication, and absorption. The score of dedication of subjects with a college or higher education was significantly higher than that of subjects with a junior college or lower education (*t* = 2.378; *P* < 0.05).

### 3.2. Correlations among Study Variables

Correlations among study variables are presented in Table 2. Extrinsic effort was negatively correlated with vigor, dedication, and absorption, while reward, POS, PsyCap, self-efficacy, hope, resilience, and optimism were positively correlated with vigor, dedication, and absorption. For overcommitment, it was not correlated with vigor, dedication, and absorption.

### 3.3. Associations of Extrinsic Effort, Reward, Overcommitment, and POS with PsyCap and Its Components

The results of hierarchical linear regression analysis on the associations of extrinsic effort, reward, overcommitment, and POS with PsyCap and its components are presented in Table 3. After adjusting for age, marital status, and education, extrinsic effort was negatively associated with optimism (*β* = −0.099; *P* < 0.01); reward had a positive association with optimism (*β* = 0.073; *P* < 0.05); overcommitment was positively associated with self-efficacy (*β* = 0.113; *P* < 0.01). POS had positive associations with PsyCap (*β* = 0.463; *P* < 0.01), self-efficacy (*β* = 0.401; *P* < 0.01), hope (*β* = 0.430; *P* < 0.01), resilience (*β* = 0.335; *P* < 0.01), and optimism (*β* = 0.435; *P* < 0.01).

### 3.4. Associations of Extrinsic Effort, Reward, Overcommitment, POS, and PsyCap and Its Components with Vigor, Dedication, and Absorption

The results of hierarchical linear regression analysis on the associations of extrinsic effort, reward, overcommitment, POS, and PsyCap and its components with vigor, dedication, and absorption are presented in Table 4. After adjusting for age, marital status, and education in block 2, extrinsic effort was negatively associated with vigor (*β* = −0.207; *P* < 0.01), dedication (*β* = −0.206; *P* < 0.01), and absorption (*β* = −0.187; *P* < 0.01); reward was positively associated with vigor (*β* = 0.072; *P* < 0.05), dedication (*β* = 0.104; *P* < 0.01), and absorption (*β* = 0.080; *P* < 0.05); overcommitment was positively associated with vigor (*β* = 0.074; *P* < 0.05), dedication (*β* = 0.089; *P* < 0.01), and absorption (*β* = 0.090; *P* < 0.01); and POS was positively associated with vigor (*β* = 0.358; *P* < 0.01), dedication (*β* = 0.361; *P* < 0.01), and absorption (*β* = 0.273; *P* < 0.01), explaining 21.9%, 23.6%, and 14.8% of the variance of the three dependent variables, respectively. In block 3's model 1, PsyCap was positively and significantly associated with vigor (*β* = 0.423; *P* < 0.01), dedication (*β* = 0.430; *P* < 0.01), and absorption (*β* = 0.280; *P* < 0.01), accounting for additional 13.0%, 13.5%, and 5.7% of the variance. In block's 3 model 2, hope was positively associated with vigor (*β* = 0.231; *P* < 0.01), dedication (*β* = 0.245; *P* < 0.01), and absorption (*β* = 0.205; *P* < 0.01); optimism was positively associated with vigor (*β* = 0.118; *P* < 0.01) and dedication (*β* = 0.124; *P* < 0.01); however, self-efficacy and resilience were not significantly associated with vigor, dedication, and absorption. These positive psychological constructs accounted for additional 13.4%, 14.0%, and 6.2% of the variance. When PsyCap and its components were added, the absolute values of regression coefficients of extrinsic effort, reward, overcommitment, and POS on vigor, dedication, and absorption were diminished or not statistically significant. Thus, PsyCap and its components could probably function as mediators in the associations of occupational stressors and POS with the three components of work engagement.

### 3.5. Mediating Roles of PsyCap and Its Components

Based on the results of hierarchical linear regression analyses in Tables 3 and 4, asymptotic and resampling strategies were used to examine the mediating roles of PsyCap and its components. As shown in Table 5, only optimism had a slightly significant mediating role in the associations of extrinsic effort with vigor (*a* × *b* = −0.012; BCa 95% CI: −0.028, −0.002) and dedication (*a* × *b* = −0.012; BCa 95% CI: −0.029, −0.002), respectively. Also, optimism slightly mediated the associations of reward with vigor (*a* × *b* = 0.009; BCa 95% CI: 0.002, 0.021) and dedication (*a* × *b* = 0.009; BCa 95% CI: 0.002, 0.022). PsyCap, hope, and resilience mediated the associations of POS with vigor, dedication, and absorption. Optimism had a significant mediating role in the associations of POS with vigor (*a* × *b* = 0.051; BCa 95% CI: 0.021, 0.084) and dedication (*a* × *b* = 0.054; BCa 95% CI: 0.011, 0.088), respectively.

## 4. Discussion

The present study evaluated the level of work engagement, using the JD-R model, explored the associations of occupational stressors (external effort, reward, and overcommitment), POS, and PsyCap and its four components (self-efficacy, hope, resilience, and optimism) with vigor, dedication, and absorption, and examined the mediating roles of PsyCap and its components among Chinese female nurses.

The average scores of vigor, dedication, and absorption of Chinese female nurses were lower than those of nurses from other countries [6, 29, 54–56] and were similar to those of Chinese nurses in another study [57] and home-visiting nurses in Japan [10] compared with the results from previous studies on work engagement in nursing workforce using the same measurement tool. Also, they were lower than those of other occupational populations, such as hospital doctors and teachers [58, 59]. As a whole, our results indicated that there was a low level of work engagement among Chinese female nurses. It is likely to be caused by the adverse work characteristics of nurses. Nurses often have to face high physical and psychological demands that can result in decreased vigor. Nevertheless, there are no adequate rewards such as job promotion, stability, respect, and income for them in the workplace. Overall, the high effort of nurses is not matched by adequate rewards, which indicates a failed social reciprocity. Thus, the negative perception of nursing work may reduce their dedication and absorption at work.

The current findings have theoretical and practical implications for the JD-R model. Work engagement is the result of the different interactions between job demands and job resources and personal resources. As job demands, extrinsic effort was negatively associated with vigor, dedication, and absorption. This finding was consistent with previous studies from different countries [26–28]. There are two possible explanations for the negative relations. One of the explanations is that, as an important occupational stressor, excessive extrinsic effort can induce burnout in workplaces, the opposite side of work engagement [58], and mental health problems such as depressive and anxious symptoms [20, 48]. Another explanation is that excessive effort or overload has negative relationships with many positive organizational behavioral outcomes, such as job satisfaction, morale, and motivation [60], which may have positive effects on work engagement. On the contrary, reward had a positive association with vigor, dedication, and absorption in this study [5, 29]. In general, extrinsic effort and reward play opposite roles in the organizational behavior and mental health of occupational populations. In the present study, compared with reward, extrinsic effort showed higher associations with vigor, dedication, and absorption. In addition, overcommitment as an internal occupational stressor may have a positive role in promoting the work engagement of nurses [61]. However, many previous studies showed that, as an internal occupational stressor, overcommitment can cause depression, anxiety, burnout, and other mental health problems in various professional populations worldwide. The results of this study confirmed that overcommitment can increase the level of work engagement, and then it may have some positive effects on various organizational behavior outcomes. Managers can not improve the job performance of workers at the expense of their mental health. Taking into account the negative impact of overcommitment on mental health across occupational populations [62], the positive effects of overcommitment on organizational behaviors should be rigorously regulated in practice. Therefore, nurse managers in China should be aware of the risk of occupational stressor for work engagement. On one hand, the managers should provide a comfortable working environment for nurses and adjust their work demands (such as working time, shift, and workload) and rewards from jobs (such as income, respect, and promotion). On the other hand, nurses should avoid excessive overcommitment by using effective strategies to cope with work tasks, such as managing time, taking on suitable promise, and concentrating on efforts.

As job resources, it was found that POS was positively associated with vigor, dedication, and absorption. Consistent results can be found in some prior studies [35, 61]. This finding indicated that POS could act as a positive resource for improving work engagement in female nurses. The reason is that POS can improve nurses' work attitudes and result in many positive organizational behavior outcomes. As a result, POS can probably increase the level of work engagement [63].

The JD-R model encompasses a personal resources component and has empirically been shown to predict work engagement. As personal resources, PsyCap has been considered as a positive resource to combat the negative outcomes of stress, burnout, and work-family conflict in workplace [40–42, 48, 64]. This study showed significantly positive associations of PsyCap, hope, and optimism with vigor, dedication, and absorption among Chinese female nurses. Only optimism slightly mediated the associations of effort and reward with vigor and dedication, respectively. The results indicated that extrinsic effort could result in a low level of vigor and dedication through reducing optimism, while job reward could result in a high level of vigor and dedication through increasing optimism among Chinese female nurses. In addition, PsyCap partially mediated the associations of POS with vigor, dedication, and absorption, which indicated that PsyCap could be a positive resource to improve the work engagement of Chinese female nurses. Nurses who have more POS are more likely to possess high level of PsyCap which can in turn increase the level of work engagement. Among the components of PsyCap, hope and optimism not only had direct effects on work engagement but also showed mediating roles in the POS-work engagement association. The most important theoretical contribution of this study is that PsyCap and its components can not only directly improve work engagement but also mediate the associations of other factors with work engagement.

The JD-R model has been supported by multiple studies in various occupations [10–13]; our findings also provide empirical support for the JD-R model to Chinese nurses' work engagement. Therefore, urgent efforts should be made to enhance work engagement from both job and personal resources for nurses' population in China. Future research should pay attention to the development strategy and measures of PsyCap in Chinese nurses. On one hand, positive psychological intervention is needed in order to enhance work engagement, especially with hope and optimism as target points [58]. On the other hand, compared with occupational stress, the effect of organizational support on the development of individual PsyCap is more powerful. Effective strategies should be applied to improve the level of POS in the workplace [37]. Hospital managers should establish supportive organizational climate for female nurses, including providing comprehensive and timely support, understanding their contribution, and wellbeing.

There were several limitations in the study. First of all, the cross-sectional design can simultaneously measure the associations of occupational stress, POS, and PsyCap and its components with work engagement, but their causal relationships cannot be determined. Second, the subjects of this study were female nurses from large general hospitals and the study did not cover nurses from other types of medical institutions, such as community health centers and nonpublic hospitals. Third, this study only controlled the confounding effects of age, marriage, and education when exploring the variable associations and more possible confounders should be investigated in further studies. Also, some other factors associated with work engagement in organizational environments, such as weekly working hours, work seniority, and organizational climate, as well as individual stressors, such as coping style and personality, should be considered in order to produce more complete results and applicative implications in further studies. Additionally, the correlations among study variables might be affected by the use of self-report measures only. Some effective process control measures such as adopting measurement tools with high reliability and validity and ensuring the anonymity of respondents were carried out to reduce common-method bias.

## 5. Conclusions

In conclusion, there is a low level of work engagement among Chinese female nurses. Using the JD-R model, extrinsic effort could reduce work engagement, while reward, overcommitment, POS, PsyCap, hope, and optimism could enhance work engagement. PsyCap, hope, and optimism could function as mediators in the associations of extrinsic effort, reward, and POS with work engagement.

## Figures and Tables

**Table 1:** Demographic characteristics of subjects and the comparisons of vigor, dedication, and absorption.

Variables	*n* (%)	Vigor Mean ± SD	Dedication Mean ± SD	Absorption Mean ± SD
Total	1016 (100)	3.21 ± 1.63	3.44 ± 1.70	2.73 ± 1.70
Age (years)				
≤30	476 (46.9)	3.07 ± 1.58	3.28 ± 1.69	2.60 ± 1.61
30–40	284 (28.0)	3.07 ± 1.64	3.32 ± 1.69	2.53 ± 1.71
>40	256 (25.2)	3.62 ± 1.65^a,*∗∗*^	3.86 ± 1.65^a,*∗∗*^	3.19 ± 1.74^a,*∗∗*^
Marital status				
Single/widowed/divorced/separated	355 (34.9)	3.22 ± 1.52	3.45 ± 1.67	2.75 ± 1.60
Married/cohabitated	661 (65.1)	3.20 ± 1.69	3.43 ± 1.71	2.72 ± 1.74
Education				
Junior college or lower	553 (54.4)	3.13 ± 1.65	3.32 ± 1.72	2.70 ± 1.66
College or higher	463 (45.6)	3.30 ± 1.60	3.58 ± 1.66^b,*∗*^	2.75 ± 1.74

SD: standard deviation.

^a^Significantly higher compared with ≤30 and 30–40 years groups.

^b^Significantly higher compared with junior college or lower group.

^*∗*^ *P* < 0.05 and ^*∗∗*^*P* < 0.01 (two-tailed).

**Table 2:** Pearson's correlation coefficients between study variables.

Variables	Vigor	Dedication	Absorption
Extrinsic effort	−0.272^*∗∗*^	−0.275^*∗∗*^	−0.232^*∗∗*^
Reward	0.227^*∗∗*^	0.254^*∗∗*^	0.208^*∗∗*^
Overcommitment	0.032	0.021	0.012
POS	0.433^*∗∗*^	0.447^*∗∗*^	0.344^*∗∗*^
PsyCap	0.549^*∗∗*^	0.566^*∗∗*^	0.399^*∗∗*^
Self-efficacy	0.449^*∗∗*^	0.459^*∗∗*^	0.321^*∗∗*^
Hope	0.525^*∗∗*^	0.540^*∗∗*^	0.393^*∗∗*^
Resilience	0.449^*∗∗*^	0.463^*∗∗*^	0.320^*∗∗*^
Optimism	0.480^*∗∗*^	0.497^*∗∗*^	0.346^*∗∗*^

POS: perceived organizational support; PsyCap: psychological capital. Pearson's correlation coefficients that are statistically significant are marked with *∗* or *∗∗* depending on their *P* value. *∗* indicates *P* value <0.05, and *∗∗* indicates *P* < 0.01 (two-tailed).

**Table 3:** Associations of extrinsic effort, reward, overcommitment, and POS with PsyCap and its components.

Variables	PsyCap		Self-efficacy		Hope		Resilience		Optimism	
	Block 1 (*β*)	Block 2 (*β*)	Block 1 (*β*)	Block 2 (*β*)	Block 1 (*β*)	Block 2 (*β*)	Block 1 (*β*)	Block 2 (*β*)	Block 1 (*β*)	Block 2 (*β*)
Age	0.158^*∗∗*^	0.113^*∗∗*^	0.181^*∗∗*^	0.143^*∗∗*^	0.134^*∗∗*^	0.093^*∗∗*^	0.135^*∗∗*^	0.101^*∗∗*^	0.088^*∗*^	0.044
Marital status	0.080^*∗*^	0.035	0.081^*∗*^	0.052	0.099^*∗∗*^	0.053	0.042	0.010	0.046	0.005
Education	0.119^*∗∗*^	0.085^*∗∗*^	0.142^*∗∗*^	0.099^*∗∗*^	0.085^*∗∗*^	0.056	0.081^*∗*^	0.058	0.101^*∗∗*^	0.081^*∗∗*^
Extrinsic effort		−0.051		−0.008		−0.069		−0.024		−0.099^*∗∗*^
Reward		0.045		0.009		0.023		0.063		0.073^*∗*^
Overcommitment		0.059		0.113^*∗∗*^		0.015		0.058		0.012
POS		0.463^*∗∗*^		0.401^*∗∗*^		0.430^*∗∗*^		0.335^*∗∗*^		0.435^*∗∗*^
*F*	11.249	53.575	15.764	40.258	6.939	42.055	7.351	26.087	5.329	49.574
Adjusted *R*^2^	0.029	0.266	0.042	0.213	0.017	0.221	0.018	0.147	0.013	0.251
Δ*R*^2^	0.032^*∗∗*^	0.239^*∗∗*^	0.045^*∗∗*^	0.174^*∗∗*^	0.020^*∗∗*^	0.206^*∗∗*^	0.021^*∗∗*^	0.132^*∗∗*^	0.016^*∗∗*^	0.241^*∗∗*^

POS: perceived organizational support; PsyCap: psychological capital.

Marital status: single/widowed/divorced/separated versus married/cohabitated. Education: junior college or lower versus college or higher.

^*∗*^ *P* < 0.05 and ^*∗∗*^*P* < 0.01 (two-tailed).

**Table 4:** Associations of extrinsic effort, reward, overcommitment, POS, and PsyCap and its components with vigor, dedication, and absorption.

Variables	Vigor				Dedication				Absorption			
	Block 1 (*β*)	Block 2 (*β*)	Block 3 (*β*)		Block 1 (*β*)	Block 2 (*β*)	Block 3 (*β*)		Block 1 (*β*)	Block 2 (*β*)	Block 3 (*β*)	
			Model 1	Model 2			Model 1	Model 2			Model 1	Model 2
Age	0.164^*∗∗*^	0.120^*∗∗*^	0.072^*∗*^	0.076^*∗∗*^	0.176^*∗∗*^	0.130^*∗∗*^	0.082^*∗∗*^	0.087^*∗∗*^	0.166^*∗∗*^	0.129^*∗∗*^	0.097^*∗∗*^	0.100^*∗∗*^
Marital status	0.101^*∗∗*^	0.050	0.035	0.034	0.110^*∗∗*^	0.059	0.045	0.043	0.097^*∗∗*^	0.057	0.048	0.045
Education	0.063^*∗*^	0.053	0.017	0.020	0.084^*∗∗*^	0.078^*∗∗*^	0.041	0.044	0.023	0.019	−0.005	−0.001
Extrinsic effort		−0.207^*∗∗*^	−0.186^*∗∗*^	−0.179^*∗∗*^		−0.206^*∗∗*^	−0.185^*∗∗*^	−0.176^*∗∗*^		−0.187^*∗∗*^	−0.173^*∗∗*^	−0.166^*∗∗*^
Reward		0.072^*∗*^	0.053	0.053		0.104^*∗∗*^	0.085^*∗∗*^	0.085^*∗∗*^		0.080^*∗*^	0.067^*∗*^	0.069^*∗*^
Overcommitment		0.074^*∗*^	0.049	0.057		0.089^*∗∗*^	0.064^*∗*^	0.073^*∗*^		0.090^*∗∗*^	0.074^*∗*^	0.082^*∗*^
POS		0.358^*∗∗*^	0.163^*∗∗*^	0.156^*∗∗*^		0.361^*∗∗*^	0.162^*∗∗*^	0.155^*∗∗*^		0.273^*∗∗*^	0.143^*∗∗*^	0.138^*∗∗*^
PsyCap			0.423^*∗∗*^				0.430^*∗∗*^				0.280^*∗∗*^	
Self-efficacy				0.073				0.059				0.024
Hope				0.231^*∗∗*^				0.245^*∗∗*^				0.205^*∗∗*^
Resilience				0.067				0.069				0.029
Optimism				0.118^*∗∗*^				0.124^*∗∗*^				0.063
*F*	8.268	46.296	75.068	55.320	10.375	52.116	84.085	62.241	7.381	29.318	36.825	27.448
Adjusted *R*^2^	0.021	0.238	0.369	0.371	0.027	0.261	0.396	0.399	0.019	0.163	0.220	0.223
Δ*R*^2^	0.024^*∗∗*^	0.219^*∗∗*^	0.130^*∗∗*^	0.134^*∗∗*^	0.030^*∗∗*^	0.236^*∗∗*^	0.135^*∗∗*^	0.140^*∗∗*^	0.021^*∗∗*^	0.148^*∗∗*^	0.057^*∗∗*^	0.062^*∗∗*^

POS: perceived organizational support; PsyCap: psychological capital.

Marital status: single/widowed/divorced/separated versus married/cohabitated. Education: junior college or lower versus college or higher.

^*∗*^ *P* < 0.05 and ^*∗∗*^*P* < 0.01 (two-tailed).

**Table 5:** Mediating roles of PsyCap and its components.

Independent variables	Mediators	Vigor	Dedication	Absorption
		*a* × *b* (BCa 95% CI)	*a* × *b* (BCa 95% CI)	*a* × *b* (BCa 95% CI)
Extrinsic effort	Optimism	−0.012 (−0.028, −0.002)	−0.012 (−0.029, −0.002)	—
Reward	Optimism	0.009 (0.002, 0.021)	0.009 (0.002, 0.022)	—
POS	PsyCap	0.196 (0.159, 0.234)	0.199 (0.165, 0.238)	0.130 (0.097, 0.164)
	Hope	0.100 (0.058, 0.144)	0.106 (0.062, 0.151)	0.089 (0.044, 0.136)
	Optimism	0.051 (0.021, 0.084)	0.054 (0.011, 0.088)	—

POS: perceived organizational support; PsyCap: psychological capital; BCa 95% CI: bias-corrected and accelerated 95% confidence interval.

Age, marital status, and education were adjusted.

## Abbreviations

POS: Perceived organizational support
PsyCap: Psychological capital
UWES: Utrecht Work Engagement Scale
ERI: Effort-Reward Imbalance
SPOS: Survey of Perceived Organizational Support
PCQ: Psychological Capital Questionnaire
VIF: Variance inflation factor
SD: Standard deviation
BCa 95% CI: Bias-corrected and accelerated 95% confidence interval.
